# Optic nerve sheath diameter measurements monitor the impact of venous sinus stenosis and surgery on intracranial pressure in NF2 meningioma patients

**DOI:** 10.1038/s41598-025-11856-4

**Published:** 2025-08-17

**Authors:** Julian Zipfel, Mykola Gorbachuk, Florian Grimm, Ulrike Ernemann, Martin Ulrich Schuhmann, Marcos Tatagiba, Isabel Gugel

**Affiliations:** 1 Department of Neurosurgery, Centre of Neurofibromatosis and Schwannomatosis, Centre for Rare Diseases, Division of Pediatric Neurosurgery, University Hospital Tübingen, Tübingen, Germany; 2 Department of Neurosurgery, University Hospital Tübingen, Tübingen, Germany; 3 Department of Diagnostic and Interventional Neuroradiology, University Hospital Tübingen, Tübingen, Germany; 4 Department of Neurosurgery, Centre of Neurofibromatosis and Schwannomatosis, Centre for Rare Diseases, University Hospital Tübingen, Tübingen, Germany

**Keywords:** Neurofibromatosis Type 2 related Schwannomatosis, Intracranial pressure, Optic nerve sheath diameter, Meningioma, Neurology, Surgical oncology, Risk factors, Neurological manifestations, Imaging techniques

## Abstract

The study aimed to evaluate intracranial pressure (ICP) in NF2-associated meningiomas using perioperative optic nerve sheath diameter (ONSD) measurements. We retrospectively analyzed bilateral ONSD in 48 NF2 patients with 90 operated intracranial meningiomas. The mean ONSD was calculated while considering factors like symptoms of increased ICP, tumor location, tumor-induced venous sinus stenosis, and Simpson grading. 403 ONSD measurements were performed over a mean follow-up of 68 ± 67 months before and 27 ± 35 months after surgery. Tumor locations included the skull base (49%), convexity (22%), falx (18%), tentorium (6%), orbit (3%), and the ventricle (2%). Significant findings included a decrease in ONSD from 6.11 ± 0.89 mm to 5.88 ± 0.91 mm postoperatively (p = 0.01), with further reduction at the last MRI (mean 5.76 ± 0.86 mm, p < 0.001). Higher preoperative ONSD was associated with venous sinus stenosis (p < 0.001) and increased ICP symptoms (p = 0.033). Postoperatively, there was a complete regression of symptoms in patients with preoperative increased ICP. Early and continual ONSD monitoring is crucial for NF2 patients with intracranial meningiomas, particularly those with venous sinus stenosis or increased ICP symptoms. Surgery that preserves the venous sinus while reducing tumor volume can lower intracranial pressure even in the long run.

## Introduction

Neurofibromatosis Type 2 related schwannomatosis (formerly known as neurofibromatosis type 2, NF2) is a tumor predisposition syndrome developed by inactivating the *NF2 gene* on chromosome 22^1^. A key feature of NF2 is the development of bilateral vestibular schwannomas (VS). Intracranial meningiomas can develop in 45–58% of patients, significantly causing morbidity and mortality^1–3^. Treatment options include a watch-and-wait strategy, functional preserving surgery, and rarely radiosurgery. Surgery is recommended for medium to large tumors, regardless of symptoms, and for all symptomatic or rapidly growing tumors that may compress critical structures like the optic nerve or brainstem. Systemic treatment with bevacizumab has not shown adequate responses for these tumors^4^. While off-label use of brigatinib has shown promise in trials^5,6^, its long-term effects are still under review. In cases of multiple meningiomas, determining when to start treatment can be difficult, especially for asymptomatic or less critically localized tumors.

In NF2 patients with intracranial meningiomatosis, the development of increased intracranial pressure (ICP) poses a significant challenging comorbidity^7^ due to a high or large-sized intracranial tumor burden, ventricle or subarachnoid spaces occlusion, or venous congestion from venous sinus compression or invasion.

Imaging techniques like magnetic resonance imaging (MRI) and computed tomography (CT) can identify specific causes of elevated ICP but cannot directly estimate it. Instead, ICP can be approximated non-invasively by measuring optic nerve sheath diameter (ONSD)^8^ using ultrasound or specialized MRI sequences^9–11^. ONSD is a reliable marker for ICP and correlates well with ICP dynamics^10,12^, often showing elevation in up to 11% of routine MRI^13^. However, monitoring ICP in NF2 patients is challenging, and venous outflow obstruction may be missed due to the slow growth of meningiomas, vague symptoms, and complicating comorbidities affecting the optic nerve or orbit.

This retrospective study aimed to evaluate ICP in intracranial NF2-associated meningiomas through ONSD measurements. We analyzed changes in ONSD before and after surgery, considering factors like symptoms/signs of increased ICP, tumor localization, tumor-induced venous sinus stenosis, and Simpson grading. Monitoring ONSD in NF2 patients with multiple meningiomas could help detect early signs of increased ICP and enable timely intervention.

## Material and methods

### Patients and treatment

The diagnosis of NF2-related schwannomatosis was confirmed in all patients by clinical evaluation using the updated diagnostic criteria^14^. A total of 48 NF2 patients with 90 operated intracranial histologically confirmed meningiomas were included in this retrospective analysis and were followed up between 2005 and 2022 at the Department of Neurosurgery and Centre of Neurofibromatosis and Schwannomatosis in Tübingen. All methods were carried out in accordance with the relevant guidelines and regulations. All experimental protocols were approved by the local ethics board of the Medical Faculty and the University Hospital of Tübingen. The need for informed consent for this retrospective analysis was waived by this committee (No 798/2022BO2, final approval date 21/12/2022).

A total of 80 surgeries were performed on 90 intracranial NF2-associated meningiomas. This included 72 surgeries for distinct meningiomas and eight surgeries performed on the same patients simultaneously. In these eight cases, the patients underwent surgery for 18 meningiomas, either located in entirely different regions or intraoperatively distinguishable lesions with differing histopathology.

Twenty-seven patients underwent single surgery. In contrast, 21 patients had two or more surgeries ranging from 2 to 8 meningiomas in different cranial areas. The 8 cases involving the resection of multiple meningiomas were excluded from comparing ONSD values and influencing factors. In these cases, it was not possible to associate any changes in ONSD with a specific location of the meningiomas, as several were treated simultaneously across different areas (e.g., falx and convexity).

**Fig. 1:**
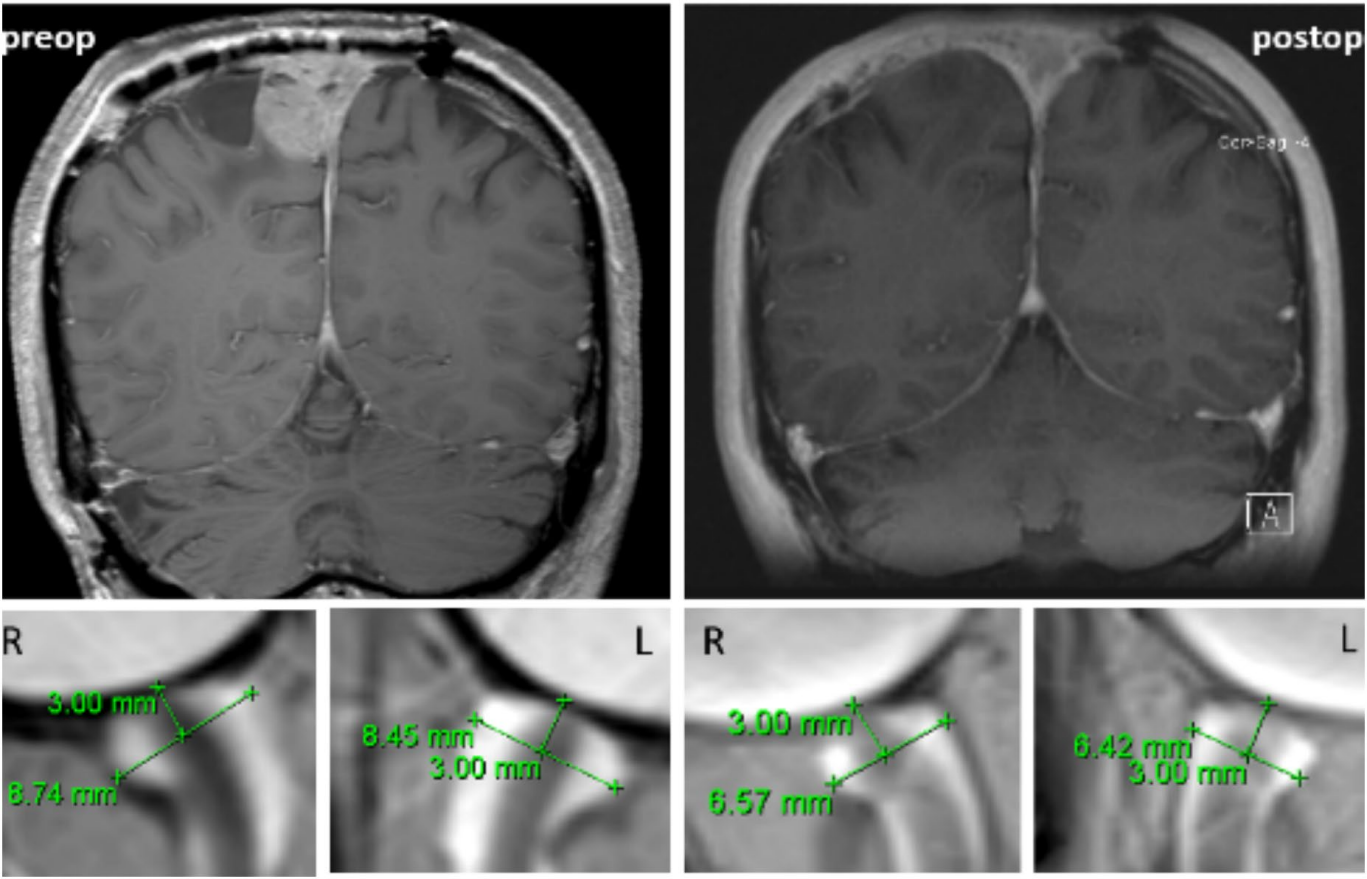
Example of an MRI-based pre- and postoperative ONSD measurement (lower row) in a study patient with a right-sided falcine meningioma and infiltration of the superior sagittal sinus with the corresponding MR imaging (upper row).

MRI-based measurements of the ONSD were conducted as previously described^9^ and as illustrated in Fig. 1.

The optic nerve appears hypointense and is surrounded by the hyperintense cerebrospinal fluid-filled optic nerve sheath. ONSD was measured in the axial plane 3 mm behind the optic nerve papilla. The massive dilatation of the optic nerve sheaths, in this case, improved postoperatively, although the values are still above 6. This highlights the importance of establishing ONSD measurements early in monitoring to assess patient-specific dynamics.

The mean values of the left and right ONSD values were calculated for further analysis and statistics. T2-weighted MRI sequences with thin slices of less than 3 mm were used at the initial and final presentations and just before and after surgery (at least 3 months before and after the procedure). A total of 403 measurements were taken by one experienced rater and independently verified for consistency by two other experienced investigators. Both internal and external MRI data sets were used. The total follow-up for ONSD measurements and clinical evaluation was 68 ± 67 months before and 27 ± 35 months after surgery.

The criteria for surgical intervention in the studied cases were based on several key factors (often combined):

- 1) The development of focal neurological deficits, seizures, or signs of increased ICP (such as papilledema, visual impairment, double vision);
- 2) Medium to large-sized tumors (> 2 cm diameter, linearly measured);
- 3) Rapid tumor growth; and.
- 4) Critical anatomical localization involving compression or invasion of critical structures, including the venous sinus, functional brain areas, cranial nerves, and the brainstem, may or may not have resulted in functional deficits. Venous sinus stenosis was defined using preoperative standard T2-weighted or T1-weighted contrast-enhanced MRI sequences, as well as available MR or CT venography and/or intraoperative findings. A venous sinus stenosis was considered potentially relevant to intracranial pressure or impair venous drainage if the compression effect within the lumen of the venous sinus was greater than 50%.

Small, uncritically localized meningiomas with a slow growth dynamic and without symptoms and no accompanying symptoms were observed.

None of the tumors had previously received radiation treatment or preoperative biopsies. All tumors were histologically confirmed as meningiomas, with the majority – 80/90 tumors – accounting for 89% classified as WHO grade I, and the remaining 10 (11%) classified as WHO grade II.

The extent of resection was evaluated according to the categories established by Simpson et al.^15^ (Table 1). Patients underwent a comprehensive pre- and postoperative clinical evaluation, including a complete medical history, a general physical assessment, a symptom-based neurological examination, and an MRI.

### Statistical analysis

We conducted the statistical analysis using SPSS Statistics 29 (IBM, NY, U.S.A.). Continuous data were presented as means (± standard deviation, ranges), while categorical data were expressed as percentages.

To assess the influence of various factors on ONSD values, measurements were taken at four key time points: the initial MRI, immediately before surgery, immediately after surgery, and at the final MRI. **A one-way repeated measures ANOVA** with Bonferroni correction was used to evaluate differences in ONSD values across these points in the overall cohort. In contrast, a **two-way repeated measures ANOVA with Bonferroni correction** examined the impact of venous sinus stenosis and ICP signs. Additionally, a multiple linear regression analyzed the relationship between ONSD values and factors such as Simpson Grading, cranial region, and the specific segment of the constricted venous sinus.

## Results

### Patients, tumors, and clinics

Detailed demographic and clinical data are summarized in Tables 1 and 2.

**Table 1:** Demographic data of 90 operated intracranial meningiomas in 48 NF2 patients.

Number of patients/tumors	48/90
Sex (Number of female/male)	26/22
Patients Age at - first NF2-related sign/symptom - the time of NF2 diagnosis - time of surgery	(mean ± SD, range) in years 18 ± 12, 0–55 21 ± 12, 1–55 34 ± 13, 9–73, n = 90
Family History with NF2-related Schwannomatosis - yes; no - no information available	number of patients (%) 9 (19%); 37 (77%) 2 (4%)
Tumor Load - further intracranial meningiomas - non-vestibular schwannoma (intracranial) - spinal ependymoma - spinal extramedullary tumors (meningiomas/schwannomas) - peripheral nerve tumors (e. g. schwannomas)	number of patients (%) 45 (94%) 21 (44%) 20 (42%) 35 (73%) 22 (46%)
Histolopathological Variants (%, n = numbers) - transitional - fibrous - meningothelial - psammoatous - angiomatous - atypical - meningothelial and angiomatous - meningothelial and psammomatous - no information available	number of tumors (%) 27 (30%) 6 (7%) 31 (34%) 11 (12%) 1 (1%) 10 (11%) 2 (2%) 1 (1%) 1 (1%)
Cranial Region Location Categories - skull base - falx - convexity - tentorium - intraorbital - intraventricular	number of tumors (%) 44 (49%) 16 (18%) 20 (22%) 5 (6%) 3 (3%) 2 (2%)
Tumor Side - left/right/median in numbers	number of tumors (%) 36 (40%)/46 (51%)/8 (9%)
Resection Extent according to Simpson Grading^15^ - I. Complete excision, including dura and bone - II. Complete excision + supposed reliable coagulation of dural attachment - III. Complete excision but insufficient dural coagulation or bone excision (not visible on MR, according to surgeon’s opinion) - IV. Incomplete excision, macroscopic residual visible (on MRI) - V. Biopsy only	number of tumors (%) 25 (28%) 26 (29%) 16 (18%) 23 (25%) 0

**SD** — standard deviation; **NF2** — Neurofibromatosis Type 2 related schwannomatosis.

**Table 2:** Preoperative and postoperative symptoms in 80 surgeries due to intracranial NF2-associated meningiomas.

	Postoperative; n = number of patients (%)
Directly preoperative number of patients (%)	At last follow-up Improved Equal Worsened
Symptomatic 47 (59%)	14 (30%) 26 (55%) 7 (15%)
Asymptomatic 33 (41%)	0 30 (91%) 3 (9%)
Focal neurological deficits * 35 (44%)	8 (23%) 21 (60%) 6 (17%)
No focal neurological deficits * 45 (56%)	0 43 (96%) 2 (4%)
Signs/symptoms of increased ICP 16 (20%) **	16 (100%) 0 0
No signs/symptoms of increased ICP ** 64 (80%)	0 62 (97%) 2 (3%)
Seizures 1 (1%)	1 (100%) 0 0
No Seizures 79 (99%)	0 77 (97%) 2 (3%)

Preoperative and postoperative status before and after surgery (evaluation at admission, and last follow-up after surgery). Preop – preoperative status/symptomatic; postop – postoperative status/symptomatic. ICP – intracranial pressure.

*Focal neurological deficits included visual impairment due to optic nerve compression/invasion, proptosis, hypacusis, gait disturbances, tinnitus, dizziness, weakness, hypesthesia, dysphagia, facial palsy, recurrent laryngeal nerve palsy) or seizures. **Signs/symptoms of increased ICP included headaches, vomiting, nausea, visual impairment, double vision, and papilledema. Preoperative signs/symptoms of increased ICP could be improved in all (16) cases.

One patient exhibited a unilateral VS. Ten patients received external off-label treatment with bevacizumab prior to surgery and due to VS. To minimize adverse events such as bleeding and wound healing disorders, systemic treatment was halted four weeks before and after surgery. None of the three patients without additional intracranial meningiomas (apart from the operated lesion) showed any other non-vestibular schwannomas besides the bilateral VS.

Of the 31 patients who underwent genetic testing, 12 patients (39%) had *NF2* truncating mutations (8 frameshift and four nonsense mutations), while 11 patients (35%) were mosaics. In the remaining eight patients, two splicing (6%), five deletions (16%), and one large genome alteration (3%) were identified in their blood DNA.

In five patients (10%), cerebrospinal fluid drainage (4 ventriculoperitoneal shunts and one endoscopic third ventriculostomy) was performed in the postoperative course due to newly developed signs of intracranial pressure.

Of the 32 (36%) meningiomas affecting the venous sinuses, 19 (59%) involved the superior sagittal sinus, 10 (31%) the cavernous sinus, two (6%) the transverse sinus, and one (3%) both the superior sagittal and confluent sinuses. A total of 67 (74%) meningiomas were completely resected (Simpson Grade I-III), while 23 (26%) were resected incompletely.

Seventy-five percent of cases with signs of increased ICP involved convexity and skull base meningiomas (each n = 6, 38%), while 19% (n = 3) and 1% (n = 1) involved falcine and tentorial meningiomas, respectively. Out of the 16 cases with signs or symptoms of increased ICP, 50% (n = 8) demonstrated a stenosis of the adjacent venous sinus. Most of these cases exhibited infiltration of the anterior or middle segment of the superior sagittal sinus, while only one tumor infiltrated each cavernous and transverse sinus.

Skull base tumors were highly (83%, n = 29) associated with focal neurological deficits, primarily due to intracranial nerve involvement, followed by falcine (6%, n = 2), intraorbital (8%, n = 3), and convexity (3%, n = 1) meningiomas, with no deficits reported in tentorial and intraventricular meningiomas. Only one patient with an operated falcine meningioma experienced seizures before surgery. In patients who experienced new postoperative seizures, two tumors were located at the falx, while the remaining tumor was found at the skull base.

Six patients exhibited postoperative complications, including cerebrospinal fluid leaks (which were manageable with a lumbar drain in all cases involving extensive intraoperative dura reconstruction), and two patients exhibited wound healing disorders, one of whom required surgical revision (this patient was under external off-label treatment with bevacizumab).

Additionally, one patient died during the postoperative outpatient period due to brain herniation after the second postoperative day and exhausted conservative and surgical (decompressive craniectomy) treatment. The exact cause of death was unclear, but there was a retrospective suspicion of silent status epilepticus or thromboembolic event. Notably, there were no postoperative bleeding events, particularly among those who externally received bevacizumab.

### Pre- and postoperative ONSD course and association with influencing factors

Data are presented as mean ± standard deviation. In the comparison analysis of the 80 performed surgeries, 2 cases had incomplete ONSD values and were excluded for further analysis. Three outliers were identified through an inspection of a boxplot. The analysis was conducted both with and without these outliers, and since the results showed no significant differences, the outliers were retained in the final dataset.ONSD values immediately after surgery were normally distributed (p > 0.05), whereas the other time points were not normally distributed, as assessed by Shapiro–Wilk’s test (p < 0.05), respectively.

Mauchly’s test of sphericity indicated that the assumption of sphericity had been violated, x^2^ (5) = 34.23, p < 0.001.

There was an increase of ONDS values from the initial MRI (mean 6 ± 1.08, 4.45–8.49 mm) to immediately before surgery (mean 6.11 ± 0.89, 4.45–8.49 mm) with a further decrease immediately after surgery (mean 5.88 ± 0.91, 4.45–7.89 mm) up to the last MRI (mean 5.76 ± 0.86, 4.55–8.03 mm).

Epsilon (ε) was 0.810, calculated by Greenhouse and Geisser (1959), and was applied to correct the one-way repeated measures ANOVA. ONSD was statistically significantly different at the different time points during the course, F (2.429, 187.069) = 7.296, p < 0.001. Post hoc analysis with a Bonferroni adjustment revealed that ONSD values at the first MRI were statistically significant to the last MRI (95%CI, 0.005 to 0.364, p = 0.04). Furthermore, ONSD values immediately before surgery were statistically significant to immediately after surgery (95%CI, 0.021 to 0.094, p = 0.01) and to the last MRI (95%CI, 0.094 to 0.467, p < 0.001). All other comparisons were not statistically significant (p > 0.05).

In the multiple regression model, Simpson grading, Cranial Region, and segment of obstructed venous sinus did not significantly predict ONSD over the four-time points, p > 0.05.

For the following analysis of influencing factors, only the 72 cases involving the resection of a meningioma in a single operation were included.

**Fig. 2:**
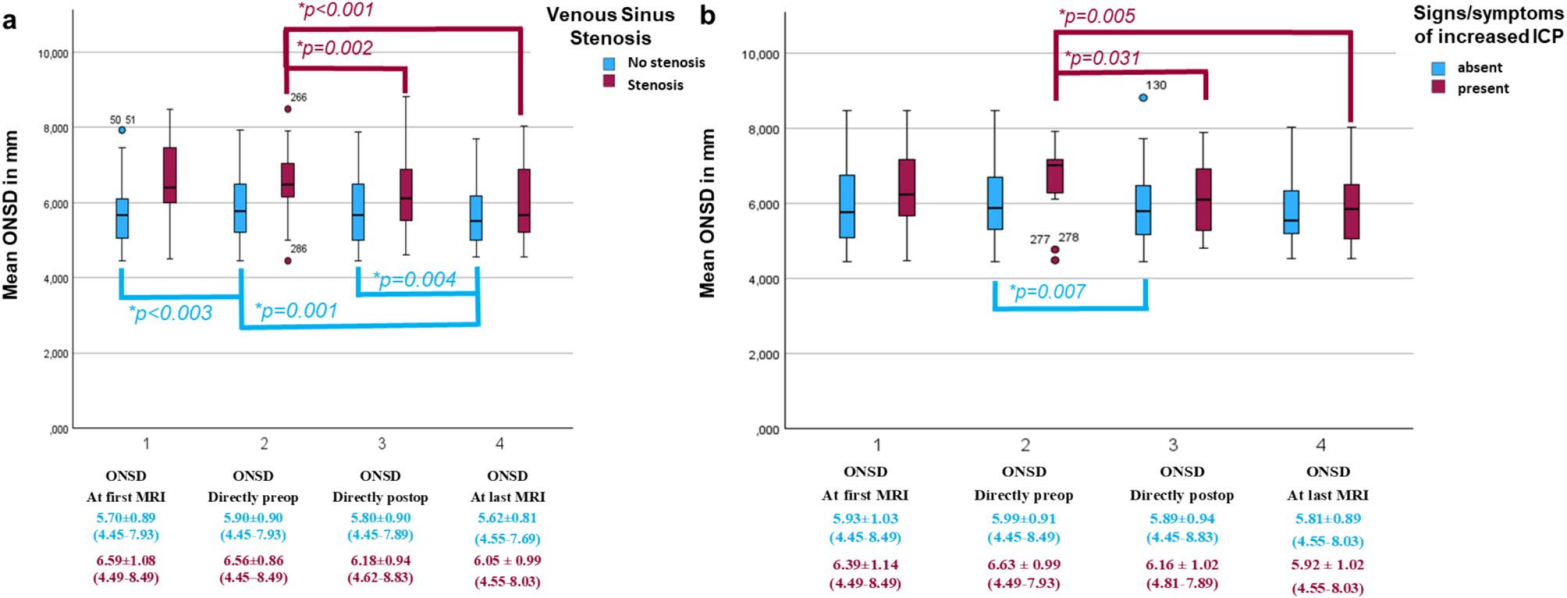
Boxplot Model of the ONSD course in the paired comparison meningioma groups: **a**) with/without venous sinus stenosis by compression or invasion, and **b**) preoperative signs/symptoms of increased ICP.

A two-way repeated measures ANOVA was run to determine the effect of venous sinus stenosis and ICP symptoms overtime on the ONSD course (ONSD at first MRI, ONSD directly preoperative, ONSD directly postoperative, ONSD at last postoperative MRI). Both comparisons for all tumors are illustrated in Fig. 2.

#### Venous sinus stenosis

The analysis of the studentized residuals confirmed normality (Shapiro–Wilk test) and the absence of outliers, as assessed by studentized residuals greater than ± 2 standard deviations. Mauchly’s test indicated sphericity (p > 0.05). A significant interaction was found between meningiomas with and without venous sinus stenosis and ONSD course, F (3,84) = 6.169, p < 0.001.

In meningiomas with venous sinus invasion or compression, significant changes in ONSD values were noted directly before and after surgery, with F (1,31) = 11.579, *p* = *0.002*, showing a mean difference of 0.381 (95% CI, 0.153 to 0.609) mm. A notable difference was also found between preoperative and last postoperative MRI values, F (1,31) = 17.061, *p* < *0.001*, mean difference of 0.510 (95% CI, 0.258 to 0.762) mm.

In contrast, meningiomas without venous sinus stenosis showed significant differences in ONSD values between the first MRI and preoperative values (F (1,55) = 9.821, *p* < *0.003*, mean difference = 0.193, 95% CI, −0.316 to −0.070), and between postoperative and last follow-up ONSD values (F(1,54) = 9.146, *p* = *0.004,* mean difference = 0.177, 95%CI, 0.060 to 0.294). A significant difference was also observed between preoperative and last MRI ONSD values (F (1,55) = 12.051, *p* = *0.001*, mean difference = 0.279, 95%CI, 0.118 to 0.440). All other comparisons were not statistically significant (p > 0.05).

#### Signs and symptoms of elevated ICP

ONSD values were normally distributed (p > 0.05) except for patients or tumors without ICP signs at the last MRI (p = 0.022), based on the Shapiro–Wilk test assessing studentized residuals. No outliers were identified, as studentized residuals greater than ± 2 standard deviations indicated. Sphericity was met for the interaction term, as evaluated by Mauchly’s test of sphericity (p > 0.05). A statistically significant interaction between meningiomas with and without increased ICP and the ONSD course was found, F (3,42) = 3.191, *p* = *0.033*.

In the meningioma group with increased ICP signs, ONSD values significantly differed directly before to after surgery, F (1,14) = 5.753, *p* = *0.031*, mean difference = 0.469 (95% CI, 0.05 to 0.89) mm. ONSD values were also statistically significantly different directly before surgery than in the last postoperative MRI, F (1,14) = 11.118, *p* = *0.005*, and the mean difference was 0.689 (95% CI, 0.246 to 1.132) mm.

On the other hand, in the meningioma group without signs of elevated ICP, ONSD values were statistically significantly different directly before surgery compared to the last postoperative MRI, F (1,62) = 7.656, *p* = *0.007*, a mean difference of 0.178 (95% CI, 0.049 to 0.307) mm. All other comparisons were not statistically significant (p > 0.05).

ICP – intracranial pressure; ONSD – optic nerve sheath diameter, in mm.

ONSD Timepoint reflects the mean ONSD at **(1)** the initial MRI, **(2)** directly before and **(3)** after surgery, and **(4)** at the last follow-up MRI. *P-values of significant comparisons are illustrated. The other comparisons were not statistically significant. Particularly in the long-term course at the last follow-up, a significant decrease compared to the preoperative mean ONSD value was seen in both groups.

In the comparison groups, 32 (36%) of the 90 operated meningiomas exhibited a venous sinus stenosis, while 58 (64%) did not. Among the 80 surgeries performed, 16 (20%) cases presented preoperative intracranial pressure symptoms, with 64 (80%) cases showing no symptoms. Signs/symptoms of increased ICP included headaches, vomiting, nausea, visual impairment, double vision, and papilledema. These signs/symptoms were explicitly distinguished from other common complaints. Signs/symptoms of increased ICP were explicitly differentiated from other common complaints, such as wound pain, known migraine or tension headaches, and potential postoperative complaints like nausea, vomiting, and headaches following surgery in the posterior fossa, which may occur due to temporary loss of cerebrospinal fluid. In tumors causing venous sinus stenosis, the immediate preoperative ONSD values were almost identical to the preoperative ONSD values. This similarity likely occurred because the surgery was performed relatively early and prophylactically to prevent further compression.

## Discussion

The management of NF2-associated meningiomas, both with and without signs of increased intracranial pressure (ICP), is complex due to the presence of numerous other tumors and pre-existing neurological impairments. Meningiomas, or tumors in general, that lead to concurrent venous sinus stenosis present particular challenges and can be difficult to treat. In NF2 patients, meningiomatosis is associated with increased morbidity and mortality.

In NF2 patients, increased intracranial pressure can result from a significant tumor burden caused by large intracranial meningiomas or schwannomas, as well as stenosis of the cerebral veins. However, the underlying causative factors remain unclear and are not fully understood, as possible compensatory mechanisms, such as the formation of collateral vessels when the sinus narrows, also contribute to the issue. Typically, problems related to intracranial pressure arise predominantly in adulthood for NF2 patients and may be related to the increasing growth dynamics of the meningiomas. Young age at diagnosis and the female sex appear favorable factors for a strong growth dynamic of NF2-associated meningiomas^16^. In our cohort, patients exhibiting signs or symptoms of increased ICP were, on average, 39 years of age (± 10, range 26–59 years) at the beginning of signs/symptoms. We demonstrated that for all patients, including those in the subgroup with preoperative sinus stenosis and ICP signs, there was a significant improvement in ONSD directly after surgery. This positive trend continued in the follow-up assessments, indicating sustained benefits from the surgical interventions.

In NF2 patients with increased ICP, surgical treatment options are limited to surgery of the presumed causative meningioma lesion, optic nerve canal decompression, depressurizing procedures via temporary acetazolamide application, CSF diversion, and lastly, hemicraniectomy. In our cohort, five patients were treated with VP-shunt or ETV in the following postoperative course due to signs of elevated intracranial pressure. Therefore, often, a combined treatment strategy in the form of systemic acetazolamide medication, CSF relief measurements, meningioma surgery (if possible), and, as the ultima ratio, hemicraniectomy should be evaluated. Interventional procedures such as venous sinus stenting should also be discussed for individual cases in an interdisciplinary team where surgical options are unavailable. White et al.^17^ investigated the effects of venous sinus stenting for tumor-induced stenosis and found it improved symptoms/signs of increased ICP. However, their investigation and review of other case series did not link this condition to NF2. The large number of meningiomas in NF2 also complicates treatment decisions.

Systemic therapeutic approaches are necessary here, mainly to control meningiomatosis. Brigatinib shows a positive response on meningeal cells and meningiomas in NF2 patients^5,6^ and should be considered mainly in NF2 patients with severe, progressive, symptomatic, and inoperable meningiomatosis at an early stage. ONSD measurement could be integrated here for therapy indication, monitoring, and dosage adjustment.

Increased values of ONSD suggest that meningiomas associated with venous sinus stenosis can lead to elevated ICP. Therefore, intervention should be before any significant compression or invasion occurs. In these cases, a combined approach that includes partial surgical removal and radiosurgery for any remaining tumor remnants may be beneficial. Additionally, it is crucial to consider the rare risk of radiation-induced malignancy, which has an absolute risk of approximately 5%^18^. This risk should be carefully weighed and thoroughly discussed with the patient to ensure an informed decision regarding the treatment options.

The measurement of the optic nerve sheath diameter (ONSD) either by ultrasound or MRI is a practical, non-invasive, quick, inexpensive, and well-tolerated tool that can be integrated easily into clinical routine and reflect the intracranial pressure^10,11^. Preliminary studies have also confirmed a good correlation between invasive and ocular fundus examinations^12^. Most studies dealing with ONSD and treatment effects were mainly related to non-tumor diseases such as hydrocephalus, and only a few were conducted on operated brain tumors^19,20^. A retrospective study of our institution investigated the ONSD on operated glioblastomas and evaluated the effect of surgery on patients with and without ICP-related symptoms. The colleagues could demonstrate a significant decrease in ONSD values after surgery and a positive correlation between ONSD and ICP-associated symptoms^19^. This effect was also seen in our NF2-meningioma cohort, with a significant decrease in ONSD values after surgery, particularly in patients with combined venous sinus stenosis and/or signs of increased ICP.

The results confirm the effectiveness of early implementation and regular monitoring of ONSD measurements via MRI or ultrasound in patients with NF2, particularly those with intracranial meningiomas that affect adjacent venous sinuses. Furthermore, this method is straightforward and non-invasive, making it practical in various neurological and neurosurgical fields and applicable to a range of conditions for assessing intracranial pressure, determining the timing for interventions, and monitoring treatment effects.

Multiple studies have been performed on children and ONSD values but less on adults; therefore, ONSD reference values are lacking. In children > 4 years, ONSD values ≥ 5 mm are considered elevated and indicate an increased ICP value. In the limited studies on healthy adults, mean ONSD values of 4.71 mm – 5.3 mm are considered“normal”^19,21,22^. In our cohort, NF2 patients with signs of elevated ICP exhibited significantly (p < 0.05) higher preoperative ONSD values (mean 6.82 ± 1.03) compared to those without (mean ONSD 6.03 ± 0.94). Whether an ONSD value of 6 should be considered“normal”remains. The individual course is decisive, so the ONSD measurement should be integrated into the early follow-up of NF2 patients. The variability in tumor burden^23^ and the duration of increased ICP^24^ contribute to the complexity of each case. The relationship between intraocular pressure^25,26^ and the optic nerve with the subarachnoid space^26^ suggests that individual cases follow unique trajectories.

The impact of venous sinus stenosis on ICP in NF2 patients remains unclear due to several factors that possibly influence it. These include tumor burden, types of diffuse meningiomas (like en plaque formations), non-tumor-related stenosis, and anatomical variations in cerebral sinuses. Additionally, diploid veins may act as collateral pathways, complicating surgical considerations, as noted by Yamashiro et al.^27^.

While it’s challenging to directly link elevated ICP symptoms to excised meningiomas, improvements in ONSD post-surgery suggest a relationship. The degree of venous sinus stenosis necessary to affect hemodynamics is not well-defined; however, some studies indicate that a stenosis of two-thirds of the lumen is clinically significant^27^. The exact role of collateral venous pathways remains uncertain, highlighting the need for standardized imaging protocols, including MRI and angiography, to facilitate further research. Overall, more prospective studies are needed to clarify the influence of venous sinus stenosis on ICP and the role of collateralization.

An important limitation of this study is the small sample size, partly due to the rare occurrence of NF2. The retrospective character of the analysis does not allow for the identification of causative factors leading to either the elevation or decrease of ONSD. Also, NF2-associated optic-orbital pathologies, such as tumor lesions in the orbit, non-tumorous eye malposition, etc., may confound ONSD. Thus, monitoring and treatment indication should be based not only on ONSD values but also on additional findings (tumor growth and size, clinical symptoms, ophthalmological evaluation), which should always be considered. In addition, up to 94% of our cohort have additional intracranial tumors, like meningiomas or schwannomas, which also influence intracranial pressure.

## Conclusions

Increased intracranial pressure (ICP) is a significant concern for NF2 patients with meningiomatosis, particularly those with meningiomas at the falx, which affects the venous sinus. These patients often present higher ONSD values before surgery. Surgical treatment of meningiomas with preservation of venous drainage can effectively reduce ICP in the long term. For this reason, early ONSD measurements via MRI or ultrasound should be implemented early in monitoring NF2 patients.

## Data Availability

All available data is included in the manuscript.
